# Evaluating the Effectiveness of a Pre-Medical Pathway Program Containing Strengths-Based Innovations

**DOI:** 10.5334/pme.2330

**Published:** 2026-08-31

**Authors:** Gianna Giacomotto, Ricardo Antillon, Esmeralda Trejo, Gerardo Moreno, Eraka Bath, Gregg J. Gold, Jennifer M. Lucero

**Affiliations:** 1 California State University Long Beach, US; 2 David Geffen School of Medicine-UCLA, US; 3 California State Polytechnic University, Humboldt, US

## Abstract

**Background & Need for Innovation:**

Summer pathway programs are designed to support students from under-resourced communities aspiring to enter healthcare professions. This study evaluated the effectiveness of a program containing strengths-based components within the UCLA Pre-Med Enrichment Program (PREP), a six-week academic enrichment initiative. PREP aims to highlight and support the individual strengths students bring to the profession while reducing psychological barriers to success.

**Goal for Innovation:**

Strengths-based components were incorporated into the 2022–2024 PREP curriculum to improve outcomes.

**Steps Taken for Development and Implementation of Innovation:**

Students participated in workshops focused on identifying and developing the strengths and experiences they bring to medicine, alongside traditional deficit-based activities emphasizing what students lack. Drawing from psychological literature, the additional curriculum was designed to shift mindsets and reduce barriers related to self-efficacy, stereotype threat, and imposter syndrome.

**Outcomes of Innovation:**

The strengths-based components yielded positive outcomes. Post-program assessments found significant reductions in imposter syndrome and stereotype threat, and increased self-efficacy. No significant changes were found in gender identity, ethnic stigma consciousness, or math identification.

**Critical Reflection on your Process:**

Strengths-based components should be considered for pathway programs and may also hold value for undergraduate medical education. Integrating curricular elements that highlight students’ strengths and promote positive self-perceptions can help reduce psychological barriers to academic and professional success. Future research should explore strategies to engage pre-medical students with low self-efficacy to help reduce attrition among aspiring medical professionals.

## Background & Need for Innovation

Students often face psychological barriers that limit academic, professional, and personal success [1,2,3]. UCLA’s Pre-Medical Enrichment Program (PREP) includes strength-based components designed to reduce these barriers. This evaluation is one of the few studies that includes psychological constructs to assess the effectiveness of such a program. Assessment of three primary psychological success-related mechanisms, self-efficacy (SE), Imposter Syndrome (IS), and stereotype threat (ST), served as outcome measures of UCLA’s strength-based portion of PREP.

Bandura’s self-efficacy theory (1993, 1994) [4] posits that an individual’s performance outcomes, experiences with success and failure, and personal beliefs in their ability to accomplish their goals will influence their levels of achievement. Self-efficacy has been demonstrated to be a significant contributor to success in various domains, including academic achievement and personal well-being [4,5]. Depending on an individual’s level of self-efficacy, performance may either be enhanced or hindered as a result of their beliefs, motivations, and actions. In medical school, higher levels of self-efficacy may serve as a protective factor, given its role in regulating stress and anxiety [4].

Also related to motivation and success is the Imposter Phenomenon, known as Imposter Syndrome (IS) [6]. IS is the feeling that one’s achievements are undeserved and that one will be exposed as a fraud. This can lead to distress and inhibit performance [5,7]. While IS can adversely impact anyone, underrepresented individuals are disproportionately affected [7]. Previous studies found that Impostor Syndrome significantly affects medical students, highlighting a peak in severity during the third year of medical school [8].

Lastly, stereotype threat is the fear of personally confirming a negative performance stereotype about one’s group in a specific domain, thus inhibiting performance. Multiple affective, cognitive, and motivational processes may underpin the effects of stereotype threat on performance [9]. The more one identifies with a negatively stereotyped group, the more susceptible an individual is to its effects [10]. However, positive role models can help mitigate the adverse impact of stereotype threat on performance [11].

Any one of these psychological concepts can affect one’s ability to succeed academically, professionally, and personally. Here, we evaluate all three within UCLA’s PREP program. PREP, updated in 2022 to include strengths-based components, is a 44-year-old program designed to increase the success of premedical students who are from medically underserved communities (MUC), including individuals from economically and/or educationally disadvantaged backgrounds.

UCLA’s PREP, for most of its existence, was similar to traditional deficit-based pathway programs that focus primarily on increasing analytical and scholastic skills. Based on personal experiences observing class based assumptions and how they affected performance and academic research in this area, and after observing the strengths-based psychological components of Olympic-level coaching such as self-efficacy and imposter syndrome, J.L., who oversees the outreach program, was inspired to change PREP to include these elements.

Since 2022, PREP has been a six-week summer academic enrichment pathway program that now includes components of a strengths-based approach designed to address the psychological barriers to success described above. Consistent with traditional medical pathway programs, PREP also continues to focus on improving students’ analytical and scholastic skills that they may be lacking before entering medical school (see Table 2).

## Goal for Innovation

At United States universities, summer pathway programs are offered to students with a strong interest in pursuing a healthcare profession [1]. These programs are designed to be academically enriching while exposing students to the medical profession. Their goal is to help students strengthen their medical school applications and provide support and mentorship that they would not otherwise have access to [1]. In this paper, we evaluate the addition of strength-based components that focus on the strengths and experiences the student brings to the profession, rather than what they are lacking. More specifically, we examine how students’ levels of self-efficacy, Imposter Syndrome, and stereotype threat were impacted by the program. While strengths-based frameworks are not new, the deliberate implementation and evaluation of this approach to pre-medical education enrichment is novel.

## Steps Taken for the Development and Implementation of Innovation

Students spent four weeks in-person in the Los Angeles metropolitan area, with the two weeks immediately prior in an online portion of the program. Once arriving, they either lived on campus (no-cost housing was provided) or had easy access to the UCLA campus.

During the on-campus portion of the program, PREP students participated in workshops, MCAT preparation courses, a health disparities project, strength-based “Mental Health is your Wealth” sessions, problem-based learning sessions, Mock Multiple Mini Interviews (MMIs,) high-fidelity medical simulations, a health-equity research methods seminar and lectures related to the medical field (Table 2).

The unique Mental Health is Your Wealth strengths-based model was introduced for the 2022–2024 PREP cohorts, with changes primarily led by the UCLA Center of Excellence and Pathways team. PREP students met weekly with a licensed psychologist to strengthen students’ self-efficacy and sense of self to reduce limiting beliefs surrounding personal achievement. The psychologist who led these sessions was chosen specifically because they work primarily with professional and college athletes who perform in high-pressure, stressful environments, similar to the mental challenges that medical and residency students encounter. Like athletes, students were provided with mental tools, strategies, and techniques to reinforce their existing mental strengths to support long-term student success.

Intentionally, a majority of PREP tutors and mentors are from backgrounds similar to the PREP students. This is important to note, as role models that students can see themselves as have been shown to effectively reduce the effects of stereotype threat and imposter syndrome, and increase self-efficacy [4,11]. UCLA David Geffen School of Medicine (DGSOM) faculty and staff led all the programs or sessions. With the 2022 strength-based revisions, physician guest speakers with backgrounds and lived experiences similar to our PREP students were added to the programto increase self-efficacy and reduce Imposter Syndrome and stereotype threat [11,12,13]. Role-model physicians from Cardiology, Family Medicine, and Pathology were intentionally featured to address these psychological barriers. Students also heard personal stories from PREP student role-model mentors (most from MUCs) about their journey to medical school.

The students in this Institutional Review Board (IRB)-24-5161 approved evaluation attended the UCLA DGSOM PREP from 2022 to 2024. A total of 107 pre-medical students attended the program. Of those, 94 completed the pre- and post-test surveys whose results are included here. The majority of the students came from California (74.7%), with 25.3% from other areas in the US (Table 1).

**Table 1:** Pre-Medical student characteristics from 2022–2024 for the University of California, Los Angeles Pre-Medical Enrichment Program (UCLA PREP) (n = 94).


UCLA PREP CHARACTERISTICS	FREQUENCY(%)

Race/Ethnicity	

African American/Black	14 (14.9%)

American/Alaskan Native	3 (3.2%)

Asian	8 (8.5%)

Caucasian/White	6 (6.4%)

Middle Eastern	8 (8.5%)

Latinx/Hispanic	40 (42.6%)

Mixed	15 (16.0%)

Age Range	

23 or lower	72 (76.6%)

24 to 30	20 (21.3%)

31 or higher	2 (2.1%)

Gender	

Female	61 (64.9%)

Male	30 (31.9%)

Non-Binary	3 (3.2%)

Academic Institution	

Community College	6 (6.4%)

Transfer	34 (36.2%)

California State University	15 (16.0%)

University of California	16 (17.0%)

Private University	11 (11.7%)

Out of State 4-year Institution	12 (12.8%)


**Table 2:** Description of UCLA PREP Sessions (2022–2024).


UCLA PREP WORKSHOPS	WORKSHOP DESCRIPTION

MCAT Preparation Course	Students review all MCAT topics* through Princeton Review. Students are also required to take two practice MCAT exams. One practice exam at the beginning and one at the end of the program.

Health Disparities Project	Students form groups and research a health disparities topic of their choice. Students are given designated hours to work on their projects. During the final week, the groups present their research findings to faculty, staff, and other student groups.

Mental Health is your Wealth	Students are guided through techniques to help empower them to succeed and reframe their self-image more positively, by a board-certified licensed psychologist.

Problem-Based Learning Sessions	Students practice their problem-based learning skills by working on 3 cases during 6 different 2-hour sessions.

Mock Multiple Mini Interviews	Students practice their interviewing skills by participating in multiple mock interviews with staff and medical student peers.

Medical Simulations	Students gain experience by observing and participating in medical simulations run by medical students at the Center for Advanced Surgical and Interventional Technology (CASIT).

Medical Lectures	Students are exposed to lectures they can expect to experience in their future medical education.


*MCAT topics include Chemistry, Physics, Biology, Biochemistry, Psychology, Sociology, and Critical Analysis and Reasoning Skills.

A pre-post, internet-based survey via Qualtrics was used to collect data from PREP students. The pretest was administered on the first day of PREP during the online portion, before the students arrived at UCLA. The post-test was administered on campus during the final week of PREP. The results of the Clance Imposter Phenomenon Scale [6], Social Identities and Attitudes Scale [14], Generalized Self-Efficacy Scale [15], and the General Academic Self-Efficacy scale [16] provided statistically testable measures of the program’s effectiveness. Additionally, focus groups were conducted with a sample of PREP students, as they provide a deeper understanding of the students’ PREP experiences and helped us further interpret the data collected [17].

Two measures of self-efficacy were administered. The 10-item Generalized Self-Efficacy Scale [9] measures an overall sense of self-efficacy, while the 10-item General Academic Self-Efficacy Scale [16] measures self-efficacy across academic domains.

The 20-item Clance Imposter Phenomenon Scale [6] is a survey used to measure the student’s feelings of imposterism.

The Social Identities and Attitudes Scale (SIAS) [14] consists of 40 questions and measures Ethnic Identity, Gender Identity, Ethnic Stigma Consciousness, Gender Stigma Consciousness, Negative Affect, and Math Identification. Our report focuses on Ethnic Identity and Negative Affect to assess general levels of stereotype threat. Ethnic Identity can be understood as how much an individual identifies with their ethnic group as part of their self-concept. Negative Affect is any anxiety or negative emotions associated with academic topics to which society has attached negative group stereotypes. Math Identification measures an individual’s beliefs about the value of, and perceived skills in math.

Descriptive statistics and scale scores were analyzed for the 94 students from 2022 to 2024 who completed PREP and the survey. All scales/subscales are composed of 5-point Likert questions. To enable comparisons, a scaled average score was computed by dividing all raw scores by the number of questions in each scale/subscale. This approach results in all scales having a minimum score of 1 and a maximum score of 5. The higher the score, the more intense the students’ feelings regarding each measurement. R version 4.4.1., and SPSS 29.01.0 were used for all analyses.

Pre-post mean scores that met normality assumptions were analyzed by paired-sample t-tests. Assumptions were checked to ensure the PREP data met statistical normality. Normality was also checked using quantile plots, despite having an adequate sample size (n = 95). There were no significant outliers in either group for Imposter Syndrome (IS). In the post-data, we saw two potential outliers with higher IS levels than most students. These two students’ high scores in IS were consistent from pre- to post-test. Because of the consistently high scores, these students were included in the analysis. Normality and outliers were checked for negative affect, general and academic self-efficacy, and assumptions were met. Ethnic Identity and Gender Stigma Consciousness did not meet normality or outlier assumptions, so the nonparametric Wilcoxon Signed Rank test was used.

## Outcomes of Innovation

See Table 3 for means, standard deviations, significance, and Cohen’s *d* effect size for all analyses. For reference, a small Cohen’s *d* = .2, moderate = .5, large = .8. Imposter Syndrome decreased significantly from pre- to post-test (*d* = .291). Regarding stereotype threat, negative affect means also decreased significantly (*d* = .360). For self-efficacy, both general self-efficacy (*d* = .492) and academic self-efficacy (*d* = .630) increased significantly. General and academic self-efficacy (n = 33) were measured for the first time in the 2024 PREP cohort with 34 students. There were no significant changes in mean ethnic identity score, gender stigma consciousness, gender identity score, or math identification score. Findings suggest that including the strengths-based components yielded positive results.

**Table 3:** Differences in scale scores after completion of UCLA PREP (2022–2024).


SCALE	PRE-SURVEY SCORE AVERAGE (SD)	POST-SURVEY SCORE AVERAGE (SD)	COHEN’S D EFFECT SIZE

Clance Imposter Phenomenon Scale*	3.56 (1.08)	3.34 (0.98)	.291

Ethnic Identity	4.19 (0.95)	4.29 (0.89)	.168

Gender Identity	3.42 (1.01)	3.53 (1.09)	.146

Ethnic Stigma Consciousness	3.25 (0.77)	3.28 (0.73)	.045

Stereotype Threat Negative Affect*	3.02 (0.86)	2.77 (0.96)	.360

Math Identification	3.57 (0.62)	3.63 (0.69)	.139

General Self-Efficacy*	3.22 (0.47)	3.39 (0.44)	.492

Academic General Self-Efficacy*	4.08 (0.77)	4.44 (0.52)	.630


*Note*: All means were standardized by dividing the true score by the number of questions in each scale. All averages are out of a 1 to 5 Likert scale. General and Academic Self-Efficacy have an N of 33. *Indicates a significant difference with *p* < 0.01.

To gather more personalized qualitative data, three focus groups with PREP alumni from 2022 to 2024 were conducted on Zoom on February 10, 11, & 12, 2025. Nine of 107 PREP alumni from those years participated. Students were recruited by e-mail, with each offered a $25 gift certificate as a participation incentive. A social psychologist conducted the interviews, with live notes taken by another research team member. Interviews were conducted in groups of two or three, lasting approximately an hour. Notes were edited manually by the note taker after each session, checked and approved by the interviewer, and then analyzed via thematic analysis (see Table 4). To determine recurring themes, notes were given to two reviewers from another university who were blind to the research aims. They were told only that the notes were from focus group participants from a premedical preparation program. Reviewers were instructed to identify any recurring themes and summarize them manually, avoiding AI tools such as ChatGPT. The reviewers did this independently. Recognizing that the limited sample may limit generalizability, the following overall themes emerged:

**Table 4:** Common Themes During PREP Focus Groups (2022–2024).


THEMES (# OF MENTIONS)	SELECTED QUOTATIONS

Increased Confidence & Clarity (33)	Yes, talking with other med-students gave me more confidence about being accepted and letting go of preconceived notions of prestigious schools. It boosted my confidence because it exposed me to people from many different walks of life.

Community & Networking (18)	The connections including friends, mentors and doctors have been the best part.

Exposure to Medicine & Admissions (20)	Admissions was also really great because you got to see a direct review of yourself as an applicant.

Motivation & Guidance (22)	Yes, PREP built confidence in knowing how to apply so once I knew what I was doing I gained more motivation. Also having the continued support from other PREP students has given me more motivation.

Challenges of the Program (17)	S1: The group project we all did. There were definitely people who did all of the work and some who did none of the work. It showed you that it happens everywhere though not just at your school. 4–6 weeks did not feel long enough.

Desire for More Hands-On Learning (6)	Sitting somewhere for three hours in the morning could be a lot sometimes. It was for MCAT studying which was very helpful but I wasn’t a big fan of the format. I would suggest maybe more hands-on activities or experiences showing what it’s like to be a doctor.

Representation & Returning to Community (8)	I want to come back to Humboldt county. I want to focus on women’s health here in Humboldt as we don’t have a lot of physicians let alone women. Our community has such a significant need for providers and I already feel so connected here Mainly, working with people like me and for immigrants is my goal. I have immigrant parents and so I want to work with first or second-gen communities.

Resources & MCAT Prep (11)	…It gave me a very expensive set of books to use for the MCAT that I wouldn’t have bought on my own. It gave me one on one time with the Admissions team at UCLA which maybe 1% of people get. 100%. There’s a lack of resources in my community and programs like PREP would be crucial for people from my community to reduce barriers.

Cohort Bonding (5)	The people and the cohort was and still is the longest lasting impact of the program. I still talk to my cohort and have made some lasting relationships which I can lean on during the admission process….

Positive Overall Experience (6)	The program has really encouraged me to continue on with my plan. It has also provided me with the necessary materials to succeed. It makes the process seem more real and becoming a doctor more real.


## What students said they got from PREP

Consistent with the Likert-scale results, PREP students reported feeling more confident than they felt before participating in the program in their ability to apply, be accepted, and succeed in medical school. A better understanding of the application process and interacting with current medical students and physicians, particularly with backgrounds and demographic characteristics similar to their own, was particularly impactful. Seeing people “like me” in those roles helped them see themselves in those roles. The program’s resources and tools made tackling the MCAT and application process feel more manageable, reducing psychological barriers and increasing motivation to apply to medical school. Students also indicated an increase in intent to serve rural and underrepresented communities.

## Strong Points of PREP

Students listed: relationships and a sense of community established during PREP, specifically connections with mentors, peers, and professionals as strong points. Many students also listed the psychologist-led strength-based sessions. Interacting with current medical students and medical professionals from diverse backgrounds was noted, along with admissions information, and MCAT preparation sessions. Focus group students said they wanted a longer program to provide greater access to multiple types of rotations and/or shadowing opportunities. This finding indicates students valued the hands-on learning opportunities.

## Areas for improvement

Students suggested: more mentors or tutors, with at least one assigned to each student; the ability to change mentors; more cohort bonding earlier in the program; and more small-group work. Also, more specialties should be represented in the program and more shadowing opportunities. Some said lectures were too long and would have preferred more MCAT preparation, with access to additional test preparation resources. Instituting a one-on-one portfolio review to strengthen their application was also noted.

All focus groups considered PREP worthwhile and would recommend it to others. The personal and professional connections they made were seen as one of the program’s most significant benefits. The qualitative results above are consistent with the quantitative results. Both emphasize the life-changing value of PREP to the students.

## Critical Reflection on the Process

A psychological evaluation of strengths-based pathway programs designed to support the success of students from MUCs through medical school has been largely undocumented. This report addresses this gap in the literature using validated psychological measures and focus groups. The updated strengths-based approach was valued by students, as evidenced by both quantitative and qualitative data. Students repeatedly reported the psychologist-led strengths-based sessions as a highlight of the program, emphasizing the value of this approach.

We suggest incorporating strengths-based components into traditional pre-med preparation programs, as was done here, to address the psychological aspects that may otherwise be missed.

Strengths-based enhancements may also be relevant to undergraduate medical education (UME). Given that PREP significantly increased general and academic SE in students with high baseline levels, incorporating a strengths-based curriculum into UME may reduce attrition for students in the early stages of pursuing a medical career.

Addressing the psychological mechanisms that often limit students from reaching their full academic potential fosters a more equitable and inclusive learning environment for all. Ultimately, this approach facilitates the development of a more diverse physician workforce to address healthcare inequities.

In conclusion, this evaluation highlights UCLA PREP’s effectiveness in reducing levels of stereotype threat and imposter syndrome while increasing general and academic self-efficacy. These outcomes highlight the importance of medical pathway programs including, and evaluating, strengths-based components. This paper offers new insights into the importance of addressing the psychological barriers that may be limiting students who are educationally and/or economically disadvantaged from performing to the best of their ability, and the role of strength-based components in addressing these issues. It also suggests that psychological theory can be utilized to support the professional and personal growth of these students, better preparing individuals to handle the rigor and challenges associated with medical school, residency, and beyond.
